# Analysis of the quinoa genome reveals conservation and divergence of the flowering pathways

**DOI:** 10.1007/s10142-019-00711-1

**Published:** 2019-09-12

**Authors:** Agnieszka A. Golicz, Ursula Steinfort, Hina Arya, Mohan B. Singh, Prem L. Bhalla

**Affiliations:** 1 Plant Molecular Biology and Biotechnology Laboratory, Faculty of Veterinary and Agricultural Sciences, University of Melbourne, Parkville, Melbourne, VIC Australia; 2 Facultad de Agronomía e Ingeniería Forestal, Pontificia Universidad Católica de Chile, Santiago, Chile

**Keywords:** Quinoa, Flowering, Comparative genomics, Genomic diversity, Evolution

## Abstract

Quinoa (*Chenopodium quinoa* Willd.) is a grain crop grown in the Andes renowned as a highly nutritious plant exhibiting tolerance to abiotic stress such as drought, cold and high salinity. Quinoa grows across a range of latitudes corresponding to differing day lengths, suggesting regional adaptations of flowering regulation. Improved understanding and subsequent modification of the flowering process, including flowering time, ensuring high yields, is one of the key factors behind expansion of cultivation zones and goals of the crop improvement programs worldwide. However, our understanding of the molecular basis of flower initiation and development in quinoa is limited. Here, we use a computational approach to perform genome-wide identification and analysis of 611 orthologues of the *Arabidopsis thaliana* flowering genes. Conservation of the genes belonging to the photoperiod, gibberellin and autonomous pathways was observed, while orthologues of the key genes found in the vernalisation pathway (FRI, FLC) were absent from the quinoa genome. Our analysis indicated that on average each *Arabidopsis* flowering gene has two orthologous copies in quinoa. Several genes including orthologues of MIF1, FT and TSF were identified as homologue-rich genes in quinoa. We also identified 459 quinoa-specific genes uniquely expressed in the flower and/or meristem, with no known orthologues in other species. The genes identified provide a resource and framework for further studies of flowering in quinoa and related species. It will serve as valuable resource for plant biologists, crop physiologists and breeders to facilitate further research and establishment of modern breeding programs for quinoa.

**Electronic supplementary material:**

The online version of this article (10.1007/s10142-019-00711-1) contains supplementary material, which is available to authorized users.

## Introduction

Quinoa (*Chenopodium quinoa* Willd.), a grain crop grown in the Andes, is receiving worldwide attention as a highly nutritious plant with gluten free and low-glycaemic index seeds (Bazile et al. 2016b; Zurita-Silva et al. 2014). Its unique tolerance to abiotic stresses such as drought, severe cold and high salinity allows quinoa to be grown even in the most adverse conditions (Hariadi et al. 2011; Jacobsen et al. 2003). In 2017, the first chromosome-scale reference genome sequence of quinoa was published, and it has provided the much-needed resource for plant biologists to design molecular breeding and crop improvement programs for quinoa (Jarvis et al. 2017).

Crop improvement programs mainly aim to breed high-yielding varieties to ensure future food security (Massawe et al. 2016; Nie et al. 2016; Peng et al. 2015). The yield of a crop plant is majorly governed by flowering process as it is the first step towards seed formation. However, current knowledge of the molecular basis of quinoa flowering is limited and the details of flowering genes and associated pathways in quinoa remain elusive (Jarvis et al. 2017). It has been reported that quinoa cultivars grown in South America from Colombia to southern Chile exhibit photoperiodic adaptation to the latitude. For example, cultivars grown close to equator, mainly in Colombia, are more photoperiod-sensitive. However, the photoperiod sensitivity is not observed in the cultivars of highlands such as Peru, Bolivia and coastal parts of Chile (Curti et al. 2016). Whether quinoa is a short-day or day-neutral plant is still under debate (Curti et al. 2014; Tapia 1979). Although, the studies of quinoa floral morphology and the effects of heat and light on its flowering have been undertaken, the understanding of the underlying genetic framework is lacking (Bertero et al. 1996; Lesjak and Calderini 2017). The genome sequence of quinoa serves as an entry point for deciphering the complex molecular pathways that contribute to floral evocation. This knowledge in turn will aid development of breeding and crop improvement programs for expanding quinoa cultivation in other parts of the world. Its ability to grow and survive in harsh conditions makes quinoa an ideal food crop choice for ensuring future food security in changing climatic conditions.

**Fig. 1:**
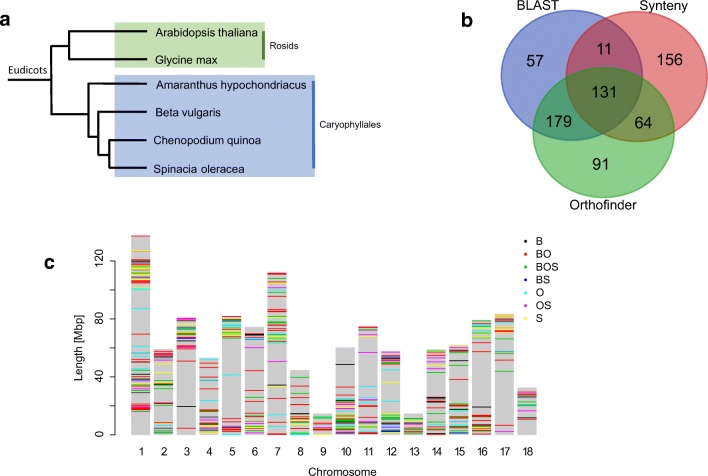
Distribution of the flowering genes identified. **a** Phylogenetic relationships between quinoa and related flowering plant species. Phylogenetic tree adapted from (Zou et al. 2017). **b** Overlap between genes identified using BLASTP only, Orthofinder and MCScanX (synteny). **c** Location of flowering genes along *C*. *quinoa* chromosomes (B – BLASTP, O – Orthofinder and S – synteny)

Flowering is governed by external and internal cues which are communicated to the plant through a complex network of flowering genes (Andrés and Coupland 2012). The flowering pathways of the model plant (Park et al. 1999) *Arabidopsis thaliana* and their corresponding flowering genes have been extensively characterised (The Arabidopsis Genome 2000). Several pathways control flowering process, and they form a complex network of genes regulating photoperiod, circadian clock function, vernalisation response, gibberellin signalling, autonomous signalling and plant ageing (reviewed in Mouradov et al. 2002b). These pathways receive signals from the environment, and fine-tune and co-ordinate them via flowering signal integrator genes (Moon et al. 2005). Finally, the output of this cross-talk leads to transition from vegetative phase to reproductive phase. However, *Arabidopsis* is a diploid species and quinoa an allotetraploid. The tetraploid quinoa genome was formed upon hybridisation of two diploid subgenomes; the A-genome from *Chenopodium pallidicaule* and the B-genome from *Chenopodium suecicum* (Brown et al. 2015; Štorchová et al. 2014; Walsh et al. 2015)*.* The hybridisation event occurred an estimated 3.3–3.6 million years ago (Jarvis et al. 2017). Further, *Arabidopsis* is a long-day flowering plant (Park et al. 1999) and quinoa ecotypes show range of photoperiodic sensitivities (Curti et al. 2016). *Chenopodium quinoa* like *Beta vulgaris*, *Chenopodium rubrum* and *Spinacia oleracea* belongs to the Amaranthaceae family (Fig. 1a) that separated from *A*. *thaliana* ~ 140 million years ago, shortly after the monocot-eudicot split (Höft et al. 2017). Hence, the differences in the chromosomal organisation of *Arabidopsis* and quinoa as well as their unique developmental characteristics will likely be reflected in genic makeup of the flowering pathways. Presence of conserved and species-specific key flowering regulators has been reported for other crop plant species like the model legume soybean and sugar beet (Jung et al. 2012; Pin Pierre et al. 2012).

We have undertaken a genome-wide analysis to identify quinoa orthologues of the *A*. *thaliana* genes, particularly those involved in flowering. As a polyploid, quinoa contains duplicate copies of most genes and those could have undergone sub- or neo-functionalisation. We identified 611 orthologues of known genes involved in flowering and performed in silico gene expression analysis across six tissues. We also identified 459 ‘orphan genes’, which are unique to quinoa and highly expressed in shoot apical meristem and flower tissue, suggesting that they could constitute a pool of quinoa-specific flowering regulators. Our study provides an essential genomic resource for functional characterisation of the quinoa flowering pathways, opening new research avenues and facilitating future efforts into breeding robust, high-yielding crop types.

## Results and discussion

### Identification of quinoa orthologues of *A*. *thaliana* flowering genes

There are 355 protein coding genes which have been identified as involved in flowering in a model plant species *A*. *thaliana* (Lin et al. 2014; Nie et al. 2016). We used a combination of sequence similarity (BLASTP search of quinoa genes against *A*. *thaliana* with 50% sequence similarity and 50% query coverage by subject thresholds and a modified Orthofinder search, see “Materials and Methods”) and collinearity (MCScanX analysis)–based approaches to identify 611 putative orthologues of 278 *A*. *thaliana* genes in quinoa (Fig. 1c). In total, 689 orthologous gene pairs were identified using both sequence similarity and collinearity, 327 gene pairs were identified using sequence similarity only and 156 gene pairs were identified using collinearity only (Fig. 1b). One-third (38.6%) of the orthologous pairs identified based on sequence similarity were also collinear, which is consistent with previous estimates (Wang et al. 2012). While only 47.3% *A*. *thaliana* genes had orthologues in quinoa which met the similarity threshold (50% similarity, 50% query coverage by subject), 78.3% of *A. thaliana* genes had orthologues identified by either BLAST search with similarity thresholds, the Orthofinder search or collinearity. The Orthofinder and collinearity searches use less stringent similarity criteria (no sequence similarity or query coverage thresholds) and the results suggest that the majority of *A. thaliana* genes have candidate orthologues in quinoa, some of them displaying considerable sequence divergence (Additional File 1). However, since the probability of functional conservation increases with sequence conservation (Joshi and Xu 2007), the subsequent analysis focuses on the 533 genes identified using identity and coverage threshold and Orthofinder searches (Table 1).

**Table 1:** Proportion of genes assigned to different categories in *A. thaliana* and quinoa

*A. thaliana* (%)	*A. thaliana* with orthologues in *C. quinoa* (%)	*C. quinoa* (%)	Pathway
37.5	36.9	36.6	Photoperiod
17.2	20.3	20.6	Flower development
15.5	11.7	12.4	Gibberellin
15.5	14.4	12.6	Vernalisation
1.1	1.8	2.3	Integrators
13.2	14.9	15.6	Other

**Fig. 2:**
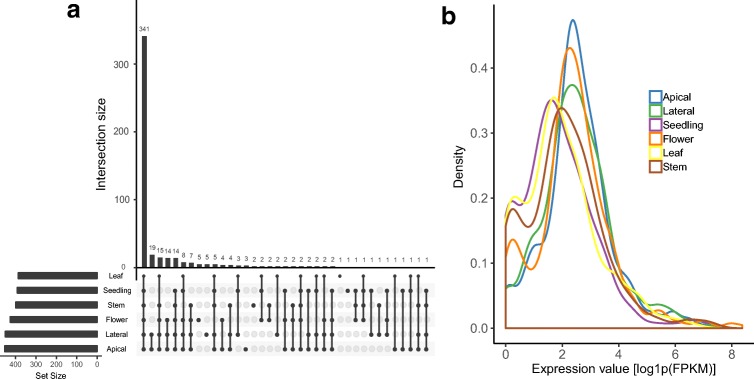
Expression of the quinoa orthologues of *A. thaliana* flowering genes. **a** Tissue distribution of expressed putative quinoa flowering genes (FPKM > 1.0). **b** Expression levels recorded for the six tissues

We investigated the expression profiles of the 533 putative quinoa flowering genes across six tissues: apical meristem, lateral meristem, flower, whole seedling, leaf and stem. In total, 90.2% of the genes had evidence of expression (FPKM > 1.0) in at least one of the tissues (Additional File 1). The mean recorded expression was 33.5 FPKM (median 7.9 FPKM). While majority of the expressed genes (70.9%) showed evidence of expression in all tissues, the highest expression points were predominantly (75.7%) recorded in the flowering-related tissues (flower and meristem) (Fig. 2a, b).

### Quinoa genome encodes genes belonging to the pathways controlling flowering in *A. thaliana*

**Fig. 3:**
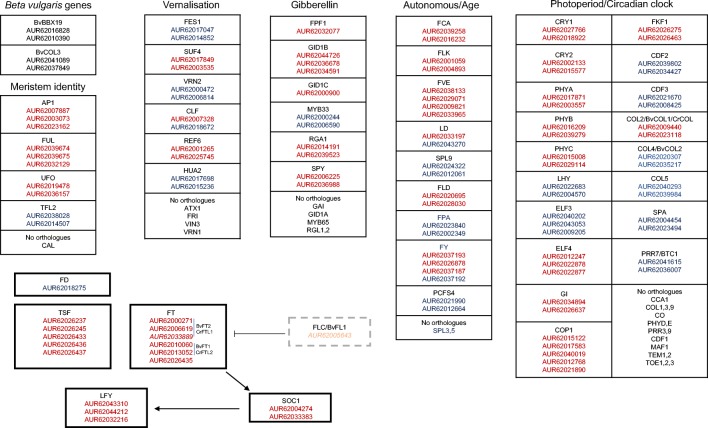
Mapping of quinoa orthologues to *A. thaliana* flowering pathways. Red font – orthologues identified using BLASTP and/or Orthofinder. Blue font – orthologues identified using Orthofinder only

In *A. thaliana*, the relationships between key flowering regulators are known. The plant senses input from multiple sources and integrates the signals received to initiate flowering. Several pathways are involved including photoperiod and circadian clock, vernalisation, gibberellin pathway and autonomous and age-related pathways, together with the floral integrators and meristem identity genes (Fig. 3). For all the pathways, orthologues of *A. thaliana* genes were identified in the quinoa genome.

#### Photoperiod and circadian clock pathway

**Fig. 4:**
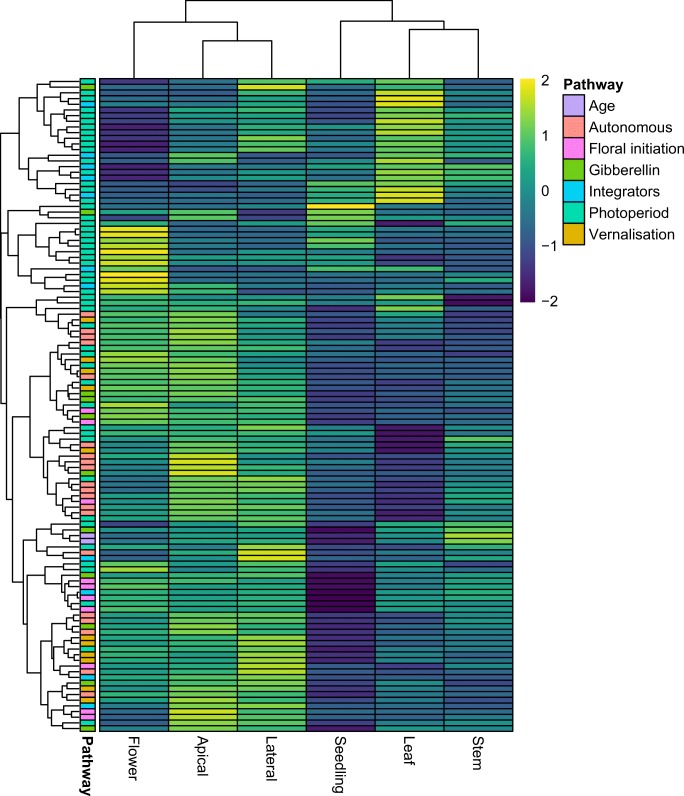
Expression patterns of the genes found in different flowering pathways.

Light is one of the main environmental regulators of flowering in plants. Plant sense day length changes and uses the cues from the environment to control the timing of the onset of flowering. The photoperiod pathway is composed of three parts: light input, circadian clock and output (Shim et al. 2017). The light signal is perceived predominantly by the blue-light-perceiving cryptochromes (CRY) and the red-light-perceiving phytochromes (PHY), and the information is integrated into the innate photoperiodic timing governed by the circadian clock genes (Shim et al. 2017). Most of the clock genes are transcriptional regulators and act as repressors, which are expressed at precise time points during the day. CIRCADIAN CLOCK ASSOCIATED 1 (CCA1) and LATE ELONGATED HYPOCOTYL (LHY) are expressed in the morning and repress evening-phased genes (Kamioka et al. 2016; Nagel et al. 2015). The PSEUDORESPONSE REGULATOR (PRR) genes are expressed during the day, repress transcription of CCA1 and LHY (Liu et al. 2016) and allow expression of evening-phased genes EARLY FLOWERING 3 (ELF3) and EARLY FLOWERING 4 (ELF4), which in turn repress PRR expression. Circadian clock is necessary for photoperiodic flowering because the key integrator gene CONSTANS (CO) is regulated by circadian clock and light signalling pathways (Shim et al. 2017). CO transcription is repressed by the family of proteins known as CYCLING DOF FACTOR (CDF) (Fornara et al. 2009), and CDF transcription is controlled by CCA1, LHY and PRRs. GIGANTEA (GI) and FLAVIN-BINDING KELCH REPEAT, F-BOX 1 form a complex which recognises CDFs as substrates for degradation. The orthologues of the key genes acting in the photoperiod pathway could be identified pointing to the pathway conservation. Orthologues of PHYA (AUR62017871, AUR62003557), PHYB (AUR62016209, AUR62039279), PHYC (AUR62015008, AUR62029114), CRY1 (AUR62027766, AUR62018922), CRY2 (AUR62002133, AUR62015577), LHY (AUR62022683, AUR62004570), ELF3 (AUR62040202, AUR62043053, AUR62009205), ELF4 (AUR62012247, AUR62022878, AUR62022877), GI (AUR62034894, AUR62026637), CDF2 (AUR62039802, AUR62034427), and CDF3 (AUR62021670, AUR62008425) were found. Most of the genes are present in two copies, which is consistent with the genome duplication history and the tetraploid nature of quinoa. The photoperiod and circadian clock genes were predominantly expressed in leaf and flower (Fig. 4).

No orthologues of CO, but six orthologues of CONSTANS-like (COL), were identified. Similarly, COL genes have been identified in two related species: *B*. *vulgaris* and *C*. *rubrum* (Chia et al. 2008; Drabešová et al. 2014). In the current analysis, counterparts of the *B*. *vulgaris* and *C*. *rubrum* and orthologues of COL2 (BvCOL1, CrCOL1 and CrCOL2) and COL4 (BvCOL2), as well as COL5 and BvCOL3, were found. In *C*. *rubrum*, a short-day plant, CrCOL1 and CrCOL2 play a role in the regulation of the circadian clock and are downregulated during the dark–light transition together with FLOWERING LOCUS T (CrFT1) (Drabešová et al. 2014). The orthologues found in quinoa may play similar roles, but their expression patterns and roles need to be confirmed by further experiments.

A close relative of quinoa, sugar beet (*B*. *vulgaris*), can behave either as an annual or biennial crop, in which flowering is triggered by long days (annual) and long days and vernalisation (biennial). Biennial genotypes evolved from annual genotypes closer to the equator to adapt to northern latitudes (Hoft et al. 2017). In *B*. *vulgaris*, the bolting locus B (Abegg 1936) is a master switch distinguishing annuals from biennials. Bolting locus B encodes a pseudoresponse regulator gene BOLTING TIME CONTROL (BTC1) (Pin Pierre et al. 2012). BTC1 is a homologue of *A. thaliana* PSEUDORESPONSE REGULATOR 7 (PRR7) involved in the circadian clock pathway. However, unlike PRR7, BTC1 responds to photoperiod and vernalisation (Pin Pierre et al. 2012). Two potential orthologues of BTC1 (AUR62041615, AUR62036007) were identified in quinoa. Another PRR7 orthologue in sugar beet, BvPRR7, which responds to both vernalisation and circadian clock, has been identified (Omolade et al. 2016), but no orthologues in quinoa were found. As quinoa is reported not to require vernalisation, potential roles of BTC1 orthologues in quinoa require further investigation.

BvBBX19 a B-box zinc finger transcription factor is another bolting time regulator, belonging to the CO-like gene family (Dally et al. 2014). BvBBX19 acts epistatically with BvBTC1 (Dally et al. 2014) and has two orthologues in quinoa (AUR62016828, AUR62010390). Both BvBTC1 and BvBBX19 act upstream of sugar beet FLOWERING LOCUS T (FT) orthologues BvFT1 and BvFT2 and are suggested to play CO function (Dally et al. 2014). Presence of both BvBBX19 and BvBTC1 orthologues in quinoa opens new avenues for the functional characterisation of these genes and detailed investigation of their roles in flowering induction in quinoa.

#### Vernalisation pathway

Many plants growing in temperate climates require vernalisation (prolonged exposure to cool temperatures) to flower (Kim et al. 2009). In contrast to cold acclimation, vernalisation requires a long-term exposure to cold (i.e. during winter) to establish flowering competency the following spring. In *A. thaliana*, the vernalisation requirement is mainly due to two dominant genes: FRIGIDA (FRI) and FLOWERING LOCUS C (FLC) (Lee et al. 1994; Michaels and Amasino 1999; Michaels and Amasino 2001). In fact, naturally occurring mutations in FRI are responsible for flowering without vernalisation in some *Arabidopsis* accessions (Johanson et al. 2000; Strange et al. 2011). The main function of FRI is upregulation of FLC transcription, which results in the vernalisation requirement (Michaels and Amasino 2001). Additional genes including FRIGIDA-ESSENTIAL 1 (FES1) and SUPPRESSOR OF FRIGIDA 4 (SUF4), both containing zinc finger domains and putative transcription factor activity, are required for FRI functionality (Kim and Sung 2014). FLC is a negative regulator of flowering and its repression is key for flowering induction. Several genes including VERNALIZATION 2 (VRN2), CURLY LEAF (CLF), VERNALIZATION 1 (VRN1) and VERNALIZATION INSENSITIVE 3 (VIN3) are involved in vernalisation mediated repression of FLC. Finally, several of the genes controlling FLC expression including the transcription factor ENHANCER OF AG-4 2 (HUA2) (Doyle et al. 2004) and histone demethylase REF6 are known to act in both vernalisation and autonomous pathways.

**Fig. 5:**
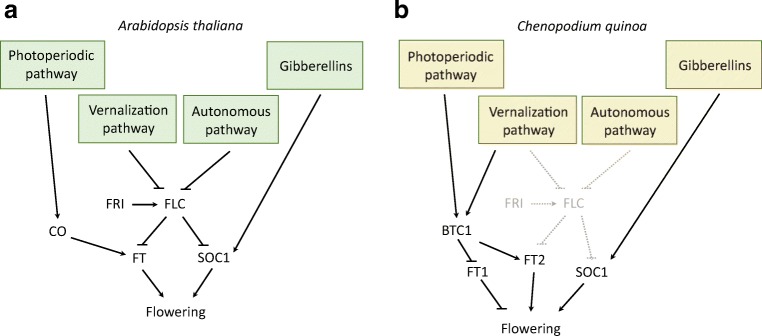
Proposed model of quinoa flowering induction as compared to *A. thaliana*. The model is based on presence and absence of *A. thaliana* orthologues in quinoa. **a** Flowering induction in *A. thaliana*. **b** Model for flowering induction in *C. quinoa*

Some of the genes involved in the vernalisation pathway of *A. thaliana* were identified in quinoa including FES1 (AUR62017047, AUR62014852), SUF4 (AUR62017849, AUR62003535), HUA2 (AUR62017698, AUR62015236), REF6 (AUR62001265, AUR62025745) and VRN2 (AUR62000472, AUR62006814). The vernalisation pathway genes were expressed predominantly in meristems (Fig. 4). A potential FLC orthologue (AUR62005643) similar to the *B*. *vulgaris* BvFL1 gene has been identified (Reeves et al. 2007). However, BLAST sequence similarity results suggest that it could be an orthologue of several FLC-related MADS box genes and not a true FLC orthologue. Additionally, BvFL1 which is the closest orthologue of FLC in sugar beet was shown not to be a major regulator of vernalisation response in biennial beets (Vogt et al. 2014), suggesting that AUR62005643 may play other roles. Although quinoa is reported not to require vernalisation to flower (Jarvis et al. 2017), its genome encodes several vernalisation-related genes. Interestingly, a similar observation was made in soybean, a short-day, vernalisation non-requiring crop plant (Jung et al. 2012). Quinoa is a frost-resistant crop grown in the Andean Altiplano (Jacobsen et al. 2007) where average temperatures during night times are at around 0 °C and frosts are common. Although many plant species in the temperate climate require vernalisation (Kim and Sung 2014), it should be noted that the two key genes (FLC, FRI) responsible for vernalisation response in *A. thaliana* are absent in quinoa. Additionally, the orthologues of genes belonging to the vernalisation pathway found in quinoa are mostly chromatin-modifying enzymes which could have acquired additional functions during evolution. The presence of several orthologues of vernalisation genes, including BTC1 and BvFL1 found in *B. vulgaris*, suggests that potential role of vernalisation should not be entirely discounted, especially in the genotypes adapted to cold climate and winter conditions. Results of the analysis performed in this study suggest that, if there are vernalisation-requiring quinoa varieties, the molecular mechanism is more likely to resemble that of sugar beet rather than the one of *A. thaliana* (Fig. 5).

#### Gibberellin pathway

Gibberellins (GAs) are plant hormones which regulate various developmental processes, including dormancy, germination, stem elongation, flowering and flower development. They function not only to stimulate growth of plant organs but also to induce developmental phase transitions (Mutasa-Göttgens and Hedden 2009). Interestingly, GA is not a universal flowering stimulus and while in some species like *Arabidopsis* GAs promote flowering, in others, for instance pea (*Pisum sativum*), increasing GA content inhibits flowering (Plackett and Wilson 2017; Reinecke et al. 2013). In *A. thaliana*, GAs have a relatively minor influence on flowering time in long-day (LD) conditions, while in short days (SD), GA pathway becomes essential (Mutasa-Göttgens and Hedden 2009). GAs activate expression of key floral integrators (SOC1, LFY and FT) (Mutasa-Göttgens and Hedden 2009). In sugar beet, the role of GAs in bolting has been reported but their involvement in floral transition remains unconfirmed (Mutasa-Göttgens et al. 2010). The genes involved in the GA pathway encode both proteins involved in GA metabolism and signal transduction factors (Mouradov et al. 2002a). GAs promote flowering by initiation of degradation of DELLA proteins including REPRESSOR OF GA (RGA), GIBBERELLIC ACID INSENSITIVE (GAI) and RGA-LIKE (RGL). GAs are perceived by soluble nuclear-localised receptor GA INSENSITIVE DWARF (GID1), which undergo conformational change allowing them to interact with the DELLA proteins. DELLA proteins in turn activate GAMYB33 expression which promotes expression of floral integrator LFY (Conti 2017; Mutasa-Göttgens and Hedden 2009). Orthologues of the key genes involved in the gibberellin pathway including GID1B (AUR62044726, AUR62036678, AUR62034591), GID1C (AUR62000900), RGA1 (AUR62014191, AUR62039523) and MYB33 (AUR62000244, AUR62006590) are present in quinoa. The results point to conservation of the gibberellin pathway in quinoa. The genes identified in this analysis can be a starting point for further molecular analysis and elucidation of the role of the gibberellin pathway in quinoa flowering as well as other biological processes.

#### Autonomous and age-related pathway

In *A. thaliana*, the autonomous pathway accelerates flowering independently of photoperiod and temperature by repression of the flowering regulator FLC (Cheng et al. 2017). Autonomous pathway genes operate mainly via chromatin epigenetic modification and post-transcriptional regulation (Cheng et al. 2017). For example FLOWERING CONTROL LOCUS A (FCA) and FPA are RNA-recognition proteins which regulate 3′-end formation, whereas FLOWERING LOCUS D (FLD) and FVE are involved in chromatin modification (Cheng et al. 2017). The key genes involved in the autonomous and age-related pathway of *A. thaliana* were identified in quinoa pointing to overall pathway conservation. The genes identified in quinoa include the following: FCA (AUR62039258, AUR62016232), FPA (AUR62023840, AUR62002349), FY (AUR62037193, AUR62026878, AUR62037187, AUR62037192), FLK (AUR62001059, AUR62004893), PCFS4 (AUR62021990, AUR62012664), FVE (AUR62038133, AUR62029071, AUR62009821, AUR62033965), FLD (AUR62020695, AUR62028030), LD (AUR62033197, AUR62043270) and SPL9 (AUR62024322, AUR62012061). The autonomous pathway genes were expressed predominantly in apical meristem (Fig. 4). Interestingly, although the key components of the autonomous pathway are conserved, their target FLC is missing from the quinoa genome. The age pathway is controlled by microRNA156 (miR156), which targets SQUAMOSA PROMOTER BINDING-LIKE (SPL) transcription factors (Wang 2014; Xing et al. 2010). In *A. thaliana*, the SPL genes can be divided into two major groups represented by SPL3 and SPL9. The SPL proteins promote flowering through activation of LEAFY (LFY), FRUITFULL (FUL) and SUPPRESSION OF CONSTANS OVEREXPRESSION 1 (SOC1) (Wang 2014). Orthologues of SPL9 but not SPL3 were identified in quinoa. The results are consistent with previous findings where SPL9 but not SPL3 orthologues were identified in monocots, suggesting that SPL3 might be evolutionarily younger (Wang and Wang 2015). Although to our knowledge no miRNA studies have been performed in quinoa, the presence of SPL9, a known miR156 target, and the evolutionary conservation of miR156 across monocots and dicots (Wang 2014) suggest possible roles of miRNAs in the control of flowering in quinoa.

#### Flowering pathway integrators

Flowering pathway integrators constitute central hubs, which integrate signals from several pathways and coordinate the precise timing of flowering. In *A. thaliana*, FLOWERING LOCUS C (FLC), SUPPRESSION OF CONSTANS OVEREXPRESSION 1 (SOC1), LEAFY (LFY) and FLOWERING LOCUS T (FT) are the main genes integrating the flowering pathways (Andrés and Coupland 2012). Additionally, TSF (TWIN SISTER OF FT) acts redundantly with FT. In the current study, six (AUR62000271, AUR62006619, AUR62033889, AUR62010060, AUR62013052, AUR62026435) and five (AUR62026237, AUR62026245, AUR62026433, AUR62026436, AUR62026437) potential orthologues of FT and TSF were identified, together with one potential orthologue of FD (AUR62018275), three of LFY (AUR62043310, AUR62044212, AUR62032216) and two of SOC1 (AUR62004274, AUR62033383). The high number of FT orthologues is consistent with previous analyses (Jarvis et al. 2017).

The putative orthologues of FT identified show divergent expression patterns (Additional File 2), suggesting possible divergence of function, specialisation or neo-functionalisation. Two of the FT homologues (AUR62006619, AUR62000271) had peak expression in leaf and two (AUR62010060, AUR62013052) had peak expression in flower. The remaining two homologues (AUR62026435, AUR62033889) did not reach the expression threshold (> 1 FPKM) in the tissues recorded. Five of the homologues are similar to the *B*. *vulgaris* BvFT1 and BvFT2 and the corresponding *C*. *rubrum* CrFTL2 and CrFTL1 genes. BvFT1 (CrFTL2) and BvFT2 (CrFTL1) have antagonistic functions. BvFT1 is a flowering repressor expressed in plants not competent to flower (Pin et al. 2010), while BvFT2 induces flowering and is upregulated by BTC1 (Pin Pierre et al. 2012). In *C. rubrum*, the CrFTL1 gene was highly upregulated after a 12-h period of darkness, resulting in flowering induction, but the function of the CrFTL2 gene was not identified (Cháb et al. 2008). The functions of the orthologues of FT genes found in quinoa require further investigation but the sequence similarity analysis suggests that they could have the antagonistic functions described for the FT genes identified in *B*. *vulgaris*.

In the genus *Chenopodium*, natural variation of the FT gene was identified owing to presence of indels in the third intron, depending on the genotype’s origin (Storchova et al. 2015). It is known that the natural variation of FT homologues facilitated regional adaptation of crop species (Wickland and Hanzawa 2015) and has been implicated in domestication of crops including rice, sunflower and soybean (Blackman et al. 2010; Ogiso-Tanaka et al. 2013; Wu et al. 2017). Further studies of FT locus diversity could help understand adaptation of quinoa to growth across a range of latitudes.

#### Master regulators of floral initiation

Meristem identity genes are activated by the upstream pathways and initiate floral development by triggering vegetative to reproductive phase transition in shoot apical meristem. The *A. thaliana* genes involved in this role include APETALA1 (AP1), CAULIFLOWER (CAL), FUL and UFO, which promote flower meristem identity and TFL, which represses floral identity (Pidkowich et al. 1999). In quinoa, orthologues of AP1 (AUR62007887, AUR62003073, AUR62023162), FUL (AUR62039674, AUR62039675, AUR62032129), UFO (AUR62019478, AUR62036157) and TFL (AUR62038028, AUR62014507) were identified, but the analysis could not detect orthologues of CAL in the quinoa genome (Fig. 3).

### Several orthologues of *A. thaliana* flowering regulators are homologue-rich in quinoa

**Fig. 6:**
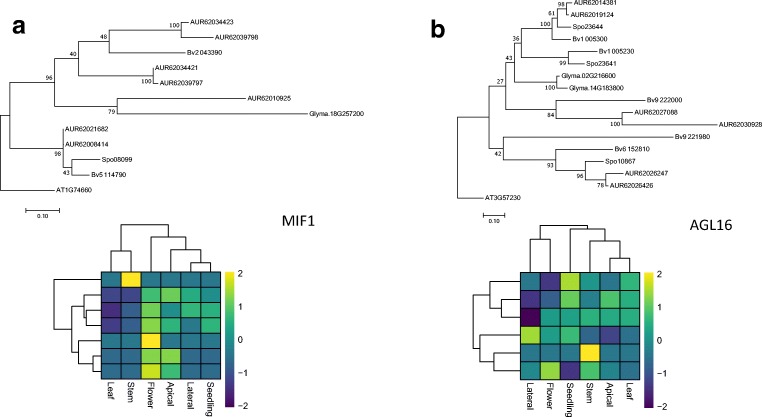
Relationships between genes present in higher copy number and their homologues in other species. **a** MIF1 gene. **b** AGL16 gene. The evolutionary history was inferred by using the maximum likelihood method based on the JTT matrix-based model. The tree is drawn to scale, with branch lengths measured in the number of substitutions per site. Bootstrapping was performed with 100 replicates. The tree was rooted using *A. thaliana* gene branch

Comparison of the number of the gene copies found in the genome relative to what is expected based on the genome duplication history can help identify genes present in the higher/lower copy number. Gene copy number expansion followed by sequence and functional diversification is considered an important mechanism of adaptation (Lespinet et al. 2002). Quinoa is a tetraploid formed upon hybridisation of two ancestral genomes, the A-genome *C*. *pallidicaule* and the B-genome diploid *C*. *suecicum*. We hypothesised that certain gene families which might have undergone expansion could contribute to the evolution and adaptation of quinoa. The median number of quinoa orthologues found for every *A. thaliana* gene is two. We searched for the genes which depart from that ratio. The maximum copy number observed was 24. The copy number counts had the following distribution: 24 copies – one gene, seven copies – two genes, six copies – two genes, five copies – 12 genes, four copies – 12 genes, three copies – 19 genes, two copies – 144 genes and one copy – 30 genes. We focused on the genes with copy number greater or equal to five (Table 2). The genes with increased copy number were involved in photoperiod pathway, flower development, gibberellin pathway, vernalisation pathway, signal integration and other functions (Table 2). The photoperiod genes include ALTERED TRYPTOPHAN REGULATION 4 (ATR4), CONSTITUTIVE PHOTOMORPHOGENIC 1 (COP1), TWIN SISTER OF FT (TSF), PHYTOCHROME A SIGNAL TRANSDUCTION 1 (PAT1) and LIGHT INSENSITIVE PHOTOPERIOD 1 (LIP1). The flower development genes include MINI ZINC FINGER (MIF1) (Fig. 6a), TRANSLATIONALLY CONTROLLED TUMOUR PROTEIN (TCTP) and CENTRORADIALIS (ATC). The two genes classified as other are AGAMOUS-LIKE 16 (AGL16) (Fig. 6b) and HEXOKINASE (HXK1). The highest number of genes with expended copy number belonged to the photoperiod pathway, suggesting that addition of new homologues to the gene repertoire of quinoa could have contributed to the adaptation of quinoa to growth across a range of latitudes (Risi and Galwey 1989). In soybean, two patterns of expression following gene duplication could be observed. Duplicated and ancestral genes either retain similar expression patterns and the expression patterns mirror phylogenetic relationships which implies conservation of function or the expression patterns diverge, which points to potential neofunctionalisation of duplicated genes (Jung et al. 2012). We investigated sequence-based phylogenetic relationships and the corresponding expression profiles of duplicated genes in quinoa and both patterns were observed. For example, duplicated homologues of MINI ZINC FINGER (MIF1) (Fig. 6a) retained similar expression patterns, whereas duplicated homologues of AGL16 (Fig. 6b) show more divergent expression patterns, which do not follow inferred sequence similarity-based phylogenetic relationships. MIF1 is involved in integration of different hormonal signals, and its constitutive overexpression results in serious developmental defects including dwarfing, reduced apical dominance and altered flower morphology (Hu and Ma 2006). Duplication of the MIF1 genes could contribute to the morphological diversity of quinoa (Risi and Galwey 1989). In *A. thaliana*, AGL16 controls flowering and its effect is dependent on the presence of FLC and FT (Hu et al. 2014). The divergent expression patterns of AGL16, as well as lack of strong putative FLC homologue in quinoa, suggests that AGL16 homologues might have acquired new functions.

**Table 2:** High copy flowering genes in quinoa. Number of copies is compared across four species (*C. quinoa*, *B. vulgaris*, *G. max*, *S. oleracea*). The values presented are the gene copy number relative to the median copy number ([total copy number compared with *A. thaliana*]/[median copy number compared with *A. thaliana* for all genes])

ID	Name	Pathway	*C. quinoa*	*B. vulgaris*	*S. oleracea*	*G. max*
AT4G31500	ATR4	Photoperiod	12	8	9	6
AT1G74660	MIF1	Flower development	3.5	2	1	0.5
AT3G16640	TCTP	Flower development	3.5	1	4	2
AT1G65480	FT	Integrators	3	2	4	4.5
AT3G57230	AGL16	Other	3	5	3	1
AT1G74670	GASA6	Gibberellin	2.5	5	6	1.5
AT1G79460	GA2	Gibberellin	2.5	1	2	0.5
AT2G21660	CCR2	Other	2.5	2	3	1.5
AT2G27550	ATC	Flower development	2.5	1	3	2.5
AT2G32950	COP1	Photoperiod	2.5	2	2	1
AT2G39540	F12 L6.20	Gibberellin	2.5	2	2	0
AT3G26744	ICE1	Vernalisation	2.5	2	2	2.5
AT4G20370	TSF	Photoperiod	2.5	0	1	1
AT4G21690	GA3OX3	Gibberellin	2.5	0	1	0
AT4G29130	HXK1	Other	2.5	1	2	2
AT5G48150	PAT1	Photoperiod	2.5	2	2	3
AT5G64813	LIP1	Photoperiod	2.5	2	2	1

### Quinoa genome encodes several hundred orphan genes potentially involved in flowering

**Fig. 7:**
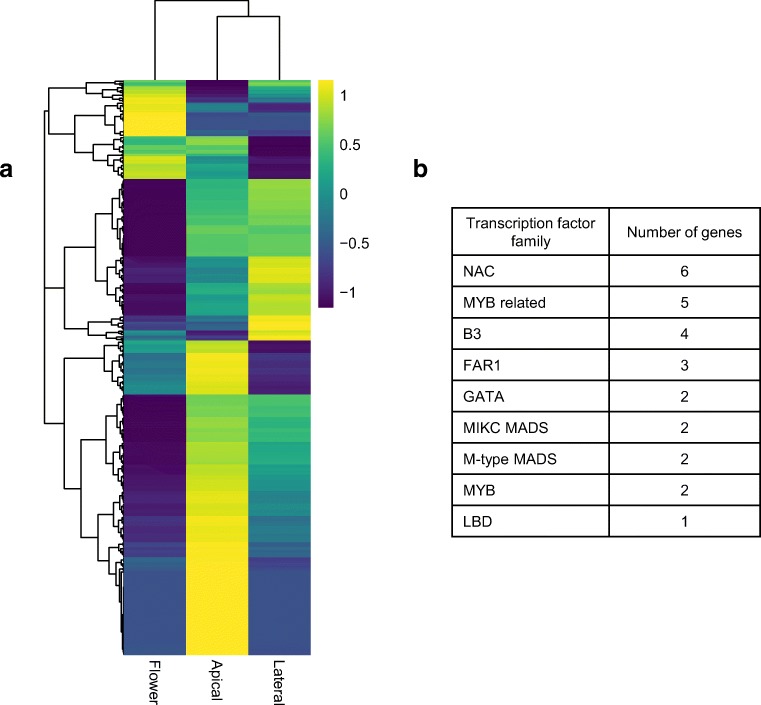
Quinoa-specific flowering regulators. **a** Tissue distribution of quinoa-specific genes expressed in flower and meristem only (FPKM>1.0). **b** Gene families represented by quinoa-specific transcription factors expressed in flower and meristem only

Orphan genes are defined as genes with protein sequences unique to a given species. Although the function of a vast majority of the newly evolved orphan genes is unknown, some were shown to be essential (Arendsee et al. 2014) and a high proportion of orphan genes in *A. thaliana* has been linked to abiotic stress tolerance (Luhua et al. 2013). Orphan genes have also been shown to be recruited to the lineage-specific structures like nodules in legumes and storage vacuoles in grasses (Arendsee et al. 2014). Quinoa is grain crop with a unique floral structure. The inflorescence is a profusely branched panicle and the flowers lack petals (Bertero et al. 1996; Bhargava et al. 2007). The genotypes display considerable variation with regard to floral morphology and duration of developmental phases leading to inflorescence formation and anthesis (Risi and Galwey 1989). Flowers of quinoa can be unisexual or hermaphrodite and vary in size and arrangement (Bhargava et al. 2007). Genes which are found uniquely in quinoa and are expressed in flowering-related tissues and structures could be contributing to the quinoa-specific floral morphology and physiology. We focused on the quinoa genes which are expressed specifically in flower and/or meristems (genes expressed in lateral meristem only were not included)—3369 genes in total. We then identified genes which are potentially *Chenopodium*-specific, i.e. genes found in *Chenopodium* species (*C*. *quinoa*, *C*. *pallidicaule* and *C*. *suecicum*) but had no orthologues in other species (*A. thaliana*, *B. vulgaris*, *Spinacia oleracea* and *Glycine max*, as identified by Orthofinder). Out of 3369 genes, 459 were *Chenopodium*-specific (Additional File3). The highest number of putative *Chenopodium*-specific flowering genes show apical meristem specific expression (269) genes (Fig. 7a). Twenty-seven of those genes were putative transcription factors, belonging to several families including NAC, B3 and MYB (Fig. 7b). The genes and especially transcription factors identified could contribute to *Chenopodium*-specific flowering characteristics.

## Conclusions

To date, the potential of quinoa as a crop plant is still untapped, and its properties remain underutilised (Bazile et al. 2016a). Our study took advantage of the recently available quinoa reference genome and identified several hundred orthologues of known flowering regulators. The results point to the overall conservation of the flowering pathways with vernalisation pathway being the most affected by the absence of orthologues of key genes. The results also point to an increased copy number of genes encoding FT and TSF genes which could contribute to the wide range of photoperiodic responses and local adaptation. The results provide a framework for the future studies of molecular mechanism of flowering in quinoa. Our identification and characterisation of flowering genes in quinoa will aid in designing studies that aim to study adaptation of quinoa to a range of latitudinal variations.

## Materials and methods

### Datasets used

*C. quinoa* (Cq_PI614886_gene_V1), *C. pallidicaule* (C_pallidicaule_gene_V1), *C. suecicum* (C_suecicum_gene_V1): http://www.cbrc.kaust.edu.sa/chenopodiumdb/

*G. max*: Gmax_275_Wm82.a2.v1, https://phytozome.jgi.doe.gov/pz/portal.html#!info?alias=Org_Gmax

*B. vulgaris*: BeetSet-2, http://bvseq.molgen.mpg.de/

*S. oleracea*: spinach_gene_v1, http://www.spinachbase.org/

*A. thaliana*: Athaliana_167_TAIR10, https://phytozome.jgi.doe.gov/pz/portal.html#!info?alias=Org_Athaliana

### Orthologous genes identification

#### BLASTP

BLASTP v2.6.0 (blastp -evalue 1e-5 -outfmt ‘6 qseqid sseqid pident length mismatch gapopen qstart qend sstart send evalue bitscore qcovs qlen slen’) was used to perform comparison of the proteomes of *C. quinoa*, *G. max*, *B. vulgaris* and *S. oleracea* against the proteome of *A. thaliana*. For each query, one best matching subject (lowest *e*-value) was retained. Then, the results were filtered to retain high scoring pairs (HSPs) with sequence similarity and query coverage by subject exceeding 50%.

#### Orthofinder

Orthofinder v2.1.2 with default parameters was used to assign the genes from all the species (*C. quinoa*, *C. pallidicaule*, *C. suecicum*, *G. max*, *B. vulgaris*, *S. oleracea*, *A. thaliana*) to orthologous gene clusters. The results were parsed to identify all the species represented by each cluster. In order to keep the most confident assignments only, a previously developed procedure was applied (Golicz et al. 2016). For the gene to be identified as orthologue, the *C. quinoa* and *A. thaliana* genes had to be found within the same cluster and constitute best matches in all vs all comparison of *C. quinoa* genes against *A. thaliana* genes.

#### Collinearity

Collinearity between the genomes of species for which pseudomolecules were available (*C. quinoa*, *G. max*, *B. vulgaris*, *S. oleracea*, *A. thaliana*) was established using MCScanX with default parameters. All vs all comparisons of proteomes were performed using DIAMOND v0.8.25 (-evalue 1e-5 -max-target-seqs 20).

### Expression analysis

The RNASeq datasets for six tissues were downloaded from NCBI Sequence Read Archive (SRA, Accessions: https://www.ncbi.nlm.nih.gov/sra/?term=SRR3938279, https://www.ncbi.nlm.nih.gov/sra/?term=SRR3938280, https://www.ncbi.nlm.nih.gov/sra/?term=SRR3938281, https://www.ncbi.nlm.nih.gov/sra/?term=SRR3938282, https://www.ncbi.nlm.nih.gov/sra/?term=SRR3938283, https://www.ncbi.nlm.nih.gov/sra/?term=SRR3938284). The reads were trimmed to remove low-quality sequence and adapter contamination using Trimmomatic v0.38 (ILLUMINACLIP:all.adapters.fa:2:30:10 LEADING:3 TRAILING:3 SLIDINGWINDOW:4:15 MINLEN:80). The reads were mapped to the *C. quinoa* genome assembly using Hisat2 v2.1.0 (--min-intronlen 20 --max-intronlen 1000). The reads mapping to the annotated genes were quantified using featureCounts v1.6.2 (-p -B -P -d 1 -D 1000 -t exon -g gene_id).

### Transcription factor identification

Transcription factors were identified by PlantTFDB ‘Transcription Factor Prediction’ tool (http://planttfdb.cbi.pku.edu.cn/prediction.php, accessed on 25.05.2018).

## Electronic supplementary material

ESM 1 (XLSX 75 kb)

ESM 2 (PDF 7 kb)

ESM 3 (XLSX 40 kb)
