# Supplementary figures and images for: Analysis of the quinoa genome reveals conservation and divergence of the flowering pathways
Source: Funct Integr Genomics. 2019 Sep 12;20(2):245–58. doi: 10.1007/s10142-019-00711-1 (PMC7018680; doi:10.1007/s10142-019-00711-1)

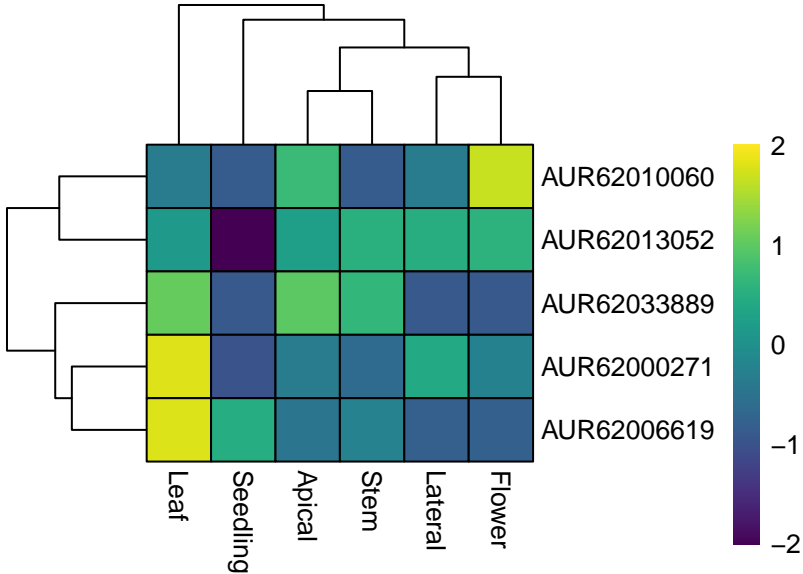

Supplement: Supplementary file 2 — (PDF 7 kb) [file 10142_2019_711_MOESM2_ESM.pdf]
